# Crocetin and crocin decreased cholesterol and triglyceride content of both breast cancer tumors and cell lines

**Published:** 2020

**Authors:** Seyed Ali Hashemi, Seyedeh Zahra Bathaie, Mohammad Ali Mohagheghi

**Affiliations:** 1 *Department of Clinical Biochemistry, Faculty of Medical Sciences, Tarbiat Modares University, Tehran, Iran*; 2 *Cancer Research Center of Cancer Institute, Imam Khomeini Hospital, Tehran University of Medical Science, Tehran, Iran*

**Keywords:** Lipid content, 4T1-induced breast tumor MDA-MB-231, MCF-7, HMGCR, Docking, Binding affinity

## Abstract

**Objective:**

Inhibition of lipid metabolism in breast cancer has been suggested as an effective approach for cancer therapy. Saffron-derived crocetin (Crt) and crocin (Cro) with the known anticancer activity, have shown hypolipidemic effect in diabetes and atherosclerosis. Here, we investigated the effect of Crt/Cro on lipid content in breast cancer.

**Materials and Methods:**

A multi-model approach involving *in vivo, in vitro *and* in silico *studies was applied. The 4T1-induced breast cancer in mice was used to investigate the effect of Crt/Cro on cholesterol (Chl) and triglyceride (TG) levels in serum and tumor tissues. The Chl/TG levels were also assessed in the cytosol of MDA-MB-231 and MCF-7 breast cancer cell lines 6, 12 and 24 hr after Crt/Cro treatment. The interaction between Crt/Cro and hydroxymethylglutaryl coenzyme A reductase (HMGCR) was also computed by docking analysis.

**Results:**

Crt reduced both serum (p=0.003) and tumor (p=0.011) Chl and TG (p=0.001) levels in mice. Cro reduced TG levels in tumor (p=0.014) and serum (p=0.002) and Chl level in tumor (p=0.013) tissues. Crt reduced both Chl and TG in MDA-MB-231 (p=0.014 and p=0.002, respectively) and MCF-7 (p=0.014 and p=0.002, respectively), after 24 h. Cro reduced both Chl and TG in MDA-MB-231 (p=0.014 and p=0.002, respectively) and MCF-7 (p=0.014 and p=0.002, respectively), after 24 h. Crt binds to the active site of HMGCR with higher affinity (ΔG^0^=-6.6 kcal/mol) than simvastatin (ΔG^0 ^=-6.0 kcal/mol).

**Conclusion:**

Crt and Cro effectively decreased Chl/TG content in the sera of tumor bearing mice, in breast tumors and breast cancer cell lines. Crt showed a higher hypolipidemic potential than Cro. *In silico* analysis indicated Crt binding in the HMGCR active site.

## Introduction

Breast cancer is one of the most prevalent cancers among women worldwide. It is estimated as the “the 5^th^ leading cause of cancer death among Iranian women” (Akbari et al., 2017 ▶). “The age-standardized rate for mortality was 14.2 per 100,000 with a mean age of 49.84 years” (Nafissi et al., 2018 ▶). Despite advances in understanding the molecular complexity of breast cancer, clinical decision that should be made for therapy still mainly relies on identifying three markers: the expression of endocrine receptors for estrogen and progesterone (ER and PR, respectively) and aberrant expression of HER2. Tumors that lack the expression of these three markers are defined as triple-negative breast cancer (TNBC) and considered the most challenging cases for breast cancer therapy. These tumors with an inherently aggressive behavior, have a relatively poor clinical outcome. Furthermore, TNBC do not benefit from the endocrine therapies (such as tamoxifen) that target the hormone receptors or HER-2 receptor (such as Herceptin) (Bianchini et al., 2016 ▶). Therefore, finding a treatment for this type of breast cancer, is very important.

Among a variety of therapies that are currently under investigation for TNBC, inhibition of mevalonate pathway by statins was reported as an effective method (Bathaie et al., 2017 ▶). Although precise biological mechanism of action is not fully elucidated, statin-induced growth inhibition and apoptosis induction in TNBC were achieved (Orlando et al., 2015 ▶), specifically by inhibition of hydroxymethylglutaryl coenzyme A reductase (HMGCR) (Bjarnadottir et al., 2013 ▶). HMGCR is the rate-limiting enzyme in the mevalonate pathway, which is responsible for synthesis of cholesterol (Chl) and some intermediates such as diphosphate isoprenoids (Goldstein and Brown, 1990 ▶). Previous studies revealed enhanced Chl synthesis in transformed cells compared to untransformed ones (Ginestier et al., 2012 ▶; Larsson, 1996 ▶). It was postulated that abundant availability of Chl precursor, acetyl-CoA, which is prepared via glycolysis, potentiates lipid (both Chl and fatty acids) synthesis in cancer cells (Biswas et al., 2012 ▶; Warburg, 1956 ▶). Simultaneously, an increase in the HMGCR activity is also another possible mechanism that promotes the upregulation of Chl synthesis (Gregg et al., 1982 ▶; Llaverias et al., 2011 ▶; Yachnin and Mannickarottu, 1984 ▶). It was reported that lipophilic statins (such as simvastatin) significantly inhibit the proliferation of breast cancer cell lines (Mueck et al., 2003 ▶). Beyond statins, isoprenoids are mevalonate-derived secondary metabolites of plants that have been introduced not only as the suppressor of HMGCR activity (Elson et al., 1999 ▶; Elson and Qureshi, 1995 ▶; Parker et al., 1993 ▶), but also as chemicals that downregulate the tumor HMGCR activity. A variety of isoprenoids and their derivatives such as farnesol, sesquiterpene, γ-tocotrienol, cyclic monoterpenes, d-limonene, perillyl alcohol and β-ionone decreases HMGCR content and consequently, induces cell cycle arrest and apoptosis in tumor cells (Elson et al., 1999 ▶; Mo et al., 2012 ▶; Parker et al., 1993 ▶).

Crocetin (Crt) and its di-gentiobiosyl derivative, crocin (Cro), are two main isoprenoids which are extracted from saffron stigma (Bolhasani et al., 2005 ▶). The cytotoxic effect of Crt and Cro in cancer cells (Abdullaev, 1994 ▶) and their anti-tumor effects in animal models (Dhar et al., 2009 ▶; Garcia-Olmo et al., 1999 ▶; Sajjadi and Bathaie, 2017 ▶) were reported. We previously showed the anticancer effects of both Crt and Cro in animal models of breast, and gastric cancers, as well as in breast and gastric cancer cell lines (Ashrafi et al., 2015 ▶; Bathaie et al., 2013 ▶; Faridi et al., 2019 ▶; Heidarzadeh et al., 2018 ▶; Hoshyar et al., 2013 ▶). Different mechanisms were reported to be involved in the Crt/Cro anticancer effects. These mechanisms include induction of conformational changes in DNA and B- to C-DNA transition by both Crt and Cro (Bathaie et al., 2007 ▶). In addition, they induce structural changes in histone H1 and decrease the H1-DNA complex formation (Ashrafi et al., 2005 ▶), which can change proteins expression in the cells. The induction of apoptosis and significant changes in the apoptotic markers like Bax/Bcl-2 ratio and caspase 9 expression, were also observed in cancer cell lines under Crt/Cro treatment (Bathaie et al., 2013 ▶; Escribano et al., 1996 ▶; Heidarzadeh et al., 2018 ▶). Cro also induced cell cycle arrest and down-regulated cyclin D1 through induction of p21 and p53 in N-nitroso-N-methylurea (NMU)-induced breast cancer in rats (Ashrafi et al., 2015 ▶).

In addition to the anticancer effects of Crt and Cro, the lipid-lowering effects of these isoprenoid compounds were reported in animal models of atherosclerosis (Gainer and Jones, 1975 ▶; He et al., 2007 ▶; Lee et al., 2005 ▶). In our previous works, we showed that administration of saffron aqueous extract (SAE) (Bathaei and Ashori, 2012 ▶), or Cro in diabetic rats decreased the levels of serum TG, total Chl, and low density lipoproteins (LDL) after 5 months (Shirali et al., 2013 ▶). Treatment of diabetic-atherosclerotic rats with Crt alone or in combination with glycine and N-acetyl cysteine (BM-92), improved their lipid profile. So that, serum Chl, TG, LDL and the atherogenic index (LDL/HDL ratio) were reduced, while HDL increased after three-months (Mahdavifard et al., 2016 ▶).

To our knowledge, no report exists in the literature about the hypolipidemic effects of Cro and Crt in cancer cells or animal model of cancer. Therefore, in the present study, we investigated the effect of Crt/Cro on lipid profile in the cytosolic fraction of TNBC and non-TNBC cell lines, as well as in tumor tissue and serum of animal models of breast cancer. In addition, using a computational analysis, we predicted the possible interaction between Crt/Cro and HMGCR as an important enzyme in the Chl synthesis.

## Materials and Methods

**Materials**

Dulbecco's modified Eagle medium (DMEM) and DMEM-F12 cell culture medium were purchased from Gibco, MD, USA. Fetal bovine serum (FBS) and penicillin-streptomycin (pen-strep) were purchased from Corning Life Sciences, Cambridge, MA, USA. Protease inhibitor cocktail (P8340), dimethyl sulfoxide (DMSO), RIPA lysis buffer (R0278), -(4,5-dimethyl-2-thiazolyl)-2,5-diphenyl-2H-tetrazolium bromide (MTT), sodium dodecyl sulfate (SDS), polyacrylamide, bis-acrylamide, triton X-100, and bovine serum albumin (BSA) were purchased from Sigma Aldrich, CA, USA. 

**Crocin and Crocetin preparation**

Cro and Crt were extracted and purified from the dried stigma of saffron, *Crocus sativus* obtained from Ghaenat L., using the methods that were previously described and registered by the authors (Patents No. 54958 and 54960 in Nov. 25, 2008, Iran, for Cro and Crt purification, respectively).

**MTT assay**

MDA-MB-231, MCF-7 and 4T1 breast cancer cell lines were purchased from the Iranian Biological Resource Center (IBRC), Tehran, Iran. MDA-MB-231 cells were cultured in DMEM-F12 and MCF-7 and 4T1 cells in DMEM. The media were supplemented with 10% FBS and 100 µg/ml of pen-strep, respectively.

We used the MTT assay (Gerlier and Thomasset, 1986 ▶) to determine the 50% inhibitory concentration of Crt and Cro in MDA-MB-231 and MCF-7. To prepare the Crt stock solution, 8 mg Crt was dissolved in 100 µl DMSO, then made up to 10 ml using cell culture media. The stock solution for Cro was prepared by dissolving 10 mg of Cro in 1 ml of proper media. The stock solutions of Cro/Crt, were filtered through 2 µm syringe filters. The cells (10×10^3^) were seeded in 96-well plates and incubated for 24 h at 37°C. After that, the media were removed and cells were washed with phosphate buffer saline (PBS). A wide range of Crt (0.1-0.8 mg/ml) or Cro (2-5 mg/ml) concentrations were used to treat the cells and cells were incubated for 24 h at 37°C. The treated cells were washed with PBS and then, incubated with 20 µl of 5 mg/ml MTT solution for 5 h. After that, the formazan crystals were dissolved in 200 µl DMSO, and then, the optical density (OD) of product was read at 570 nm, using a plate-reader (BioTek Instruments, Inc., Winooski, VT, USA). The experiment was performed in triplicate for each Cro or Crt concentration.

**Cell treatments**

The MDA-MB-231 and MCF-7 cells were seeded in 10 cm^2 ^plates (2×10^6 ^cells/well) and incubated at 37°C to achieve 60-70% confluency. Then, cells were washed with phosphate buffered saline (PBS). MDA-MB-231 cells were treated with 8-10 ml of media containing Crt (0.7 mg/ml) or Cro (4 mg/ml). MCF7 cells were treated with the same volume of media containing Crt (0.8 mg/ml) or Cro (3.5 mg/ml). To prepare the Crt-enriched media, first, Crt was dissolved in 100 µl DMSO and then, solubilized in the desired media and filtered; so that, the concentration of DMSO in the media was 1%. To prepare the Cro containing media, Cro was weighed and dissolved in the media and filtered through 2 µm syringe filters. Cells were cultured in Crt/Cro-containing media and incubated for 6, 12 or 24 h at 37°C in a CO_2_ incubator. Then, the cells were rinsed with PBS, harvested by trypsin and stored at -80°C.

**Animals and treatment**

To investigate the effects of Crt/Cro on *in vivo* lipid profile, the 4T1-induced breast cancer was established in Balb/c mice. Female 6-8 week-old Balb/c mice were purchased form Pasteur Institute, Karaj, Iran. Animals were fed on a routine diet and water *ad libitum*. After acclimation, animals were inoculated with 10^6^ 4T1 cells (in DMEM) per animal into their left flank. Then, tumor growth was monitored in animals. When tumors were palpable, the mice were encaged into three groups, 5 in each group of control, Crt and Cro. Animals were under intraperitoneal injection of either 150 µl of PBS, 150 mg/kg body weight Cro (dissolved in PBS) or 100 mg/kg body weight Crt (dissolved in DMSO and then in PBS; final DMSO concentration was less than 1%), once a week, for four weeks. Then, the mice were sacrificed under diethyl ether anesthesia by cardiac puncture. The blood was drawn and the tumor tissue was collected and stored at –80°C. The experiments were carried out according to the guidelines for the Care and Use of Laboratory Animals prepared by the Tarbiat Modares University.

**Isolation of cytosolic fraction**

The cells prepared from the last step, were re-suspended in 50 mM HEPES pH 7.4, 10 mM NaCl, 5 mM MgCl_2_, and 0.1 mM EDTA supplemented with protease inhibitor cocktail (Sigma Aldrich, CA, USA) and was lysed by three strokes of sonication (10 s each) by a sonicator (Misonix 3000, Farmingdale, NY, USA). The lysates were centrifuged at 14000×*g *for 20 min (4C), and the supernatant (cytosol) and pellet (membrane) were collected and stored at -80°C for further experiments (Maclellan et al., 2005 ▶). 

**Western blotting**

The concentration of protein in cytosolic and membrane-enriched fractions, prepared in the fractionation step, were determined (Bradford, 1976 ▶). Samples of both fractions with the same protein content, were subjected to 12% SDS-polyacrylamide gel electrophoresis. The proteins separated on SDS-PAGE gel, were transferred on PVDF membrane (Bio-Rad Laboratories, Inc., Hercules, CA, USA), then, probed with mouse polyclonal anti-human β-actin antibody (Ab) (Sigma Aldrich, CA, USA). After washing with TBS-T (, Tris buffered saline containing 1% Tween-20), blots of proteins were incubated with horse reddish peroxidase (HRP)-conjugated rabbit-anti-mouse β-actin secondary Ab (Sigma Aldrich, CA, USA). The ECL kit (RPN 2232, ECL, Amersham, Pharmacia Biotech, US) was used for immunodetection. Relative band densities were quantified by densitometry using Image J software (1.46r, US National Institutes of Health, USA).

**Lipids assessment**

Chl and TG were measured in the cytosolic fraction of the cell lines and tumor tissues, as well as the serum samples from mice with 4T1-induced breast cancer, using Chl and TG assay kits (Pars Azmoon, Karaj, Iran). The concentration of Chl was calculated based on a standard curve of Chl concentration, prepared using gradually increasing concentrations of pure Chl powder (Merck, Darmstadt, Germany). The TG levels of different groups were compared, as a percentage ratio of the control group.

**Molecular docking analysis**

Molecular docking was performed using AutoDock Vina (version 1.1.2) (Trott and Olson, 2010) to investigate the interaction between Cro and Crt and HMGCR, ER, PR and HER-2. For this purpose, the PDB file of Crt (CID: 5281232) and Cro (CID: 5281233) were obtained from PubChem. The following X-ray crystal structures were obtained from protein data bank (PDB) and used for docking analysis:

- Complex of catalytic portion of human HMGCR with simvastatin (SIM) (a clinically well-known inhibitor of HMGCR) (PDB ID: 1HW9).
- Human ER ligand-binding domain in complex with 17-β-estradiol (PDB ID: 1ERE).
- Progesterone-bound human PR ligand-binding domain (PDB ID: 1A28).
- Extracellular domain of human HER2 complex with Herceptin Fab (PDB ID: 1N8Z).

The original ligands including: simvastatin, 17-β-estradiol, progesterone and Herceptin were removed from the related X-ray crystal structure complex by Pymol (version 1.7.4.5). PDB files of the ligand-free X-ray crystals were imported into Auto Dock Tools (ADT), polar hydrogens and Kollman charge were added and PDBQT format of the protein was saved. Then, Crt was imported as the ligand into the ADT and the Gasteiger charges were computed. The rigid root and the rotatable bonds were defined and the torsion tree was determined, so that the maximum active torsions for the ligand was 6 bands. Grid maps of 100×100×100 points with a grid-point spacing of 0.375A˚ were generated using Auto Grid tool. Docking commands were applied under exhaustiveness equal to 400. As a result of analysis, 20 binding conformers of Crt to HMGCR were arranged in order of binding energy strength (kcal/mol). The first conformer with the minimum binding energy was selected and visualized in three dimensional (3D) by Pymol (version 1.7.4.5). The Ligplot (version 1.4.5) was used to determine the amino acids involved in the interaction with this conformer of Crt in two dimensional (2D) view.

Furthermore, we compared Crt/Cro with simvastatin, 17-β-estradiol, progesterone and Herceptin, regarding their binding with HMGCR, ER, PR and HER2, respectively. For this purpose, the best binding conformers of Crt/Cro (with the minimum binding energy), obtained from docking analysis, were selected and separately superposed with the pre-mentioned X-ray crystal structure complexes and introduced as: i to iv. The overlapping of Crt and simvastatin, 17-β-estradiol, progesterone and Herceptin in the active sites of HMGCR, ER, PR and HER2, were visualized by Pymol in 3D. To optimize the docking condition, and make the docking analysis more reliable, we re-docked simvastatin with HMGCR. Then, the best resulting conformer of simvastatin, was superposed with the pre-mentioned X-ray crystal structure complex of the catalytic portion of human HMGCR with simvastatin. The binding energy of the best conformers was calculated. 

**Statistical analysis**

SPSS software version 24.0 was used for statistical analysis. Comparison between groups in the *in vitro*, and *in vivo* experiments, was performed by one-way ANOVA. A p<0.05 was considered statistically significant. All data are expressed as mean±SD of at least three independent repeats.

## Results

**MTT assay**

**Figure 1:**
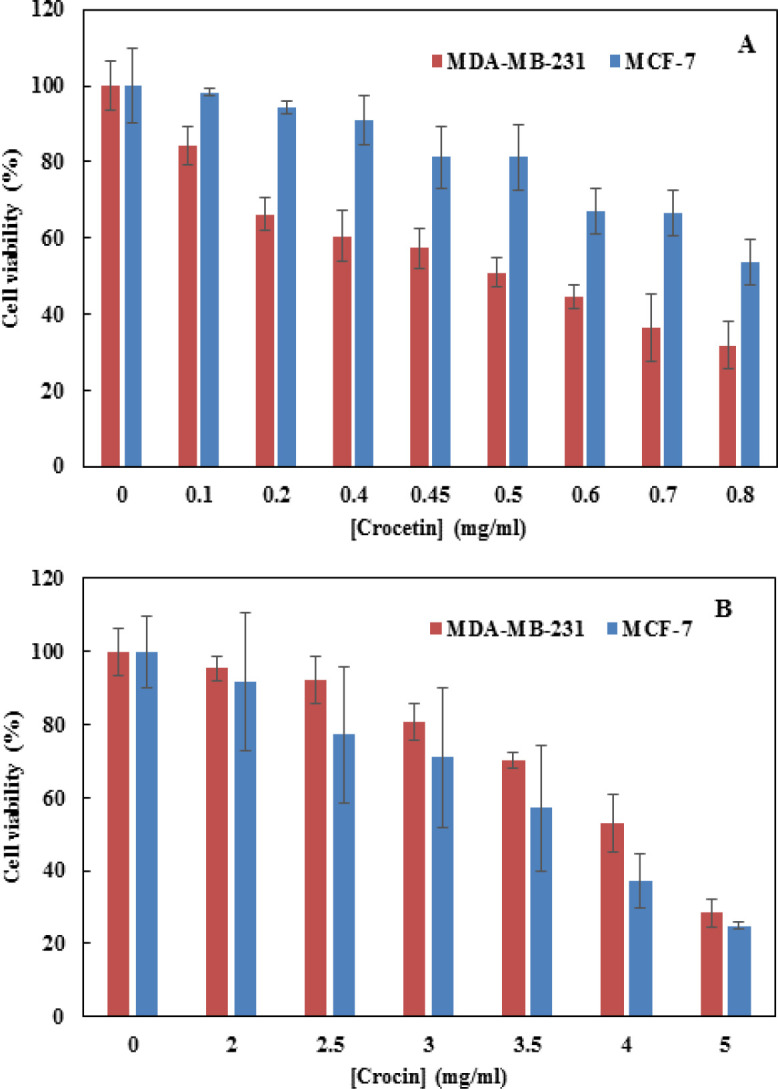
The effect of Crt (A) and Cro (B) on cell viability in MDA-MB-231 and MCF-7. The cell viability was evaluated by MTT assay after24 h treatment with 0.1-0.8 mg/ml Crt and 2-5 mg/ml of Cro. Data are presented as mean±SD, for three independent experiments

Figures 1A and 1B show the results of the MTT assay done after treatment of the mentioned cells with 0.1- 0.8 mg/ml Crt (A) or 2 to 5 mg/ml Cro (B) at 24 h, indicating the dose-dependent changes in the viability of both cell lines. Based on the data presented in Figure 1A, the IC50 of Crt was estimated 0.7 and 0.8 mg/ml for MDA-MB-231 and MCF-7, respectively. In Figure 1B, the IC50 of Cro was estimated 4 and 3.5 mg/ml for MDA-MB-231 and MCF-7, respectively. These concentrations were used in all other experiments.

**The effects of Crt/Cro on Chl level**

**Figure 2:**
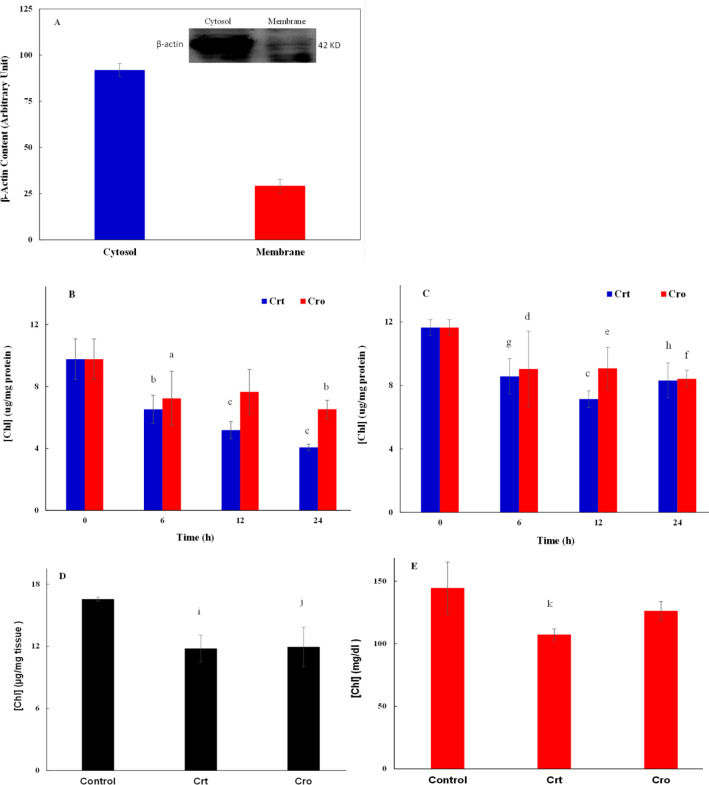
The effect of Crt/Cro on Chl levels in *in vitro* and *in vivo *models of breast cancer

Figure 2A is the representation of β-actin protein density in the cytosolic and membrane fractions of MCF-7 cells, obtained by western blotting (the inset of Figure). It shows that β-actin density in the cytosolic fraction was dramatically higher than that in the membrane fraction, indicating the good quality of the separation procedure. Thus, in the following steps, the concentrations of Chl and TG were determined in the cytosolic fractions.

Figures 2B and 2C show the Chl levels in the cytosolic fraction of MDA-MB-231 and MCF-7 cell lines treated with desired concentrations of Crt or Cro for 24 h. The results showed that treatment of both cell lines with Crt/Cro significantly reduced cytosolic Chl in a time-dependent manner. The differences in Chl between both Crt/Cro treated cell lines and the untreated one, were statistically significant at 6, 12 and 24 hr.

Figures 2D and 2E demonstrate the results of Chl assessment in the tumor tissues and sera of 4T1-induced breast cancer Balb/c mice with or without Cro/Crt treatment. The results indicate that Chl levels in both breast cancer tumors and sera of mice treated with Crt significantly reduced compared to the control mice. However, Chl reducing effect of Cro was only significant in the tumors of the treated mice. The comparison between Crt and Cro showed that Crt was more effective than Cro with respect to lowering Chl in both tumors and serum of mice, as well as the cytosol of the breast cancer cell lines studied here.

(A) The estimates of β-Actin content in the cytosolic and membrane fractions that were obtained by Image J analysis of the protein band densities obtained by western blotting and shown in the inset.

(B) and (C) Chl concentration in the cytosolic fraction of cells, prepared from MDA-MB-231 and MCF-7 cell lines treated with Crt (0.7 and 0.8 mg/ml) or Cro (4 and 3.5 mg/ml) and incubated for 0, 6, 12 and 24 h, respectively. Data are obtained from three independent experiments and presented as mean±SD.

(D) and (E) The results of Chl assessment in tumor tissues and sera of 4T1-induced breast cancer Balb/c mice, that were treated once a week, with either Crt (100 mg/kg BW) or Cro (150 mg/ kg BW) for up to 4 weeks, respectively. Data are presented as mean±SD. Chl; Cholesterol, Crt; Crocetin, Cro; Cro.

Significant differences between treated groups and the control are shown by different letters, which are defined as follows: a, p=0.017; b, p=0.002; c, p=0.000, d, p=0.032; e, p=0.035; f, p=0.007; g, p=0.010; h, p=0.005; i, p=0.011; j, p=0.013; and k, p=0.030.

**Figure 3:**
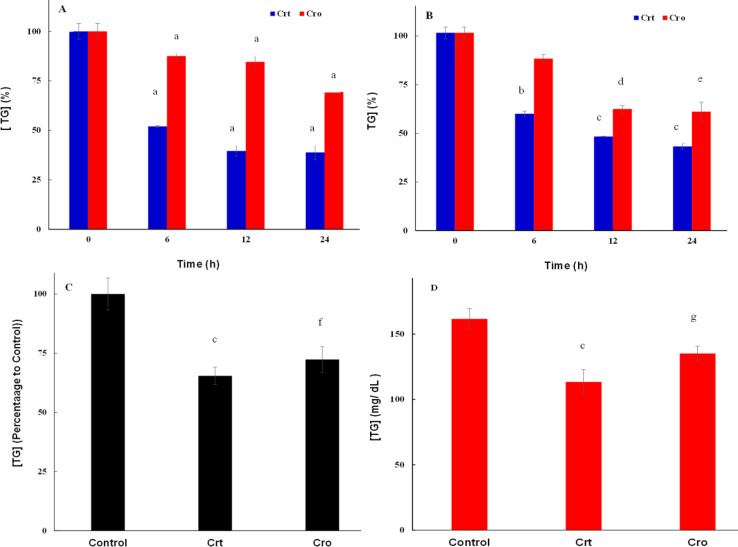
The effect of Crt/Cro on TG levels in *in vitro* and *in vivo *models of breast cancer

(A) and (B) The TG concentrations in the cytosolic fractions of MDA-MB-231 and MCF-7 cell lines treated with Crt (0.7 and 0.8 mg/ml, respectively)/Cro (4.0 and 3.5 mg/ml, respectively) at 0, 6, 12 and 24 h. The data are presented as mean±SD of three independent experiments.

(C) and (D) the results of TG assessment in tumor tissues and sera of 4T1-induced breast cancer Balb/c mice, that were treated once a week, with either Crt (100 mg/kg BW) or Cro (150 mg/kg BW) for 4 weeks, respectively. Data are presented as mean±SD.

Significant differences between treated groups and the control are shown by different letters, which are defined as follows: a, p=0.000; b, p=0.012; c, p=0.001; d, p=0.019; e, p=0.015; f, p=0.002; and g, p=0.014. TG; Triglyceride, Crt; Crocetin, Cro; Cro.

**The effects of Crt/Cro on TG level**

Figure 3A shows TG levels in the cytosolic fraction of MDA-MB-231 and MCF7 cell lines treated with Crt or Cro for up to 24 h. The results showed that treatment of both cell lines with Crt/Cro significantly reduced the cytosolic TG in a time-dependent manner. Figures 3B and 3C indicate the results of TG assessment in tumor tissues and sera of 4T1-induced breast cancer tumor Balb/c mice, treated with either Crt or Cro after for 4 weeks. The results indicated that TG levels in the sera of mice treated with either Crt or Cro significantly reduced compared to the control mice.

**Computational simulation of Crt/Cro interaction with HMGCR**

**Figure 4:**
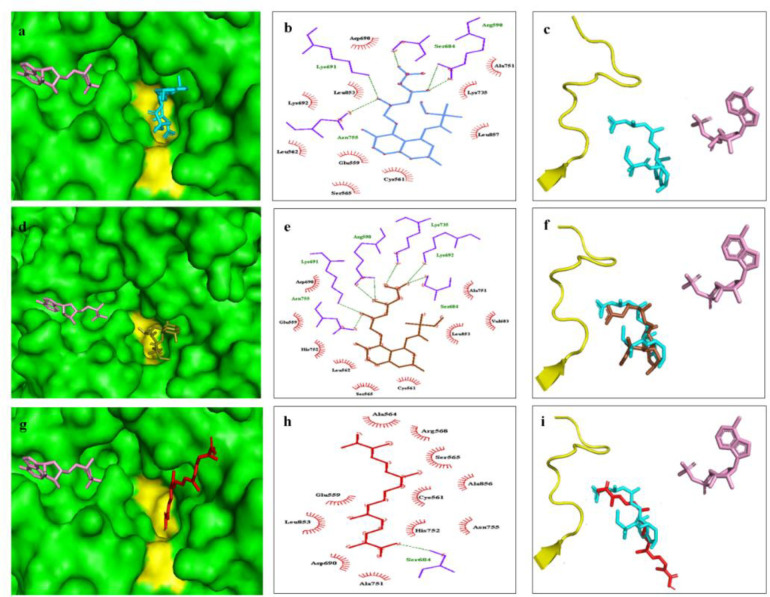
Comparison of binding sites of SIM and Crt in HMGCR. The 3D representations are shown in Figures a, d and g, and the 2D representations are shown in Figures b, e and h. Spatial location of SIM, nicotinamide adenine dinucleotide phosphate (NADP) and cis-loop in crystal structure of complex of SIM and HMGCR are shown in Figure 3a. Superposition of SIM binding mode in the crystal structure of SIM- HMGCR complex with SIM conformer (obtained from docking analysis) and Crt are shown in Figures c, f and i, respectively. The 2D views demonstrate amino acid residues of HMGCR contributing to hydrogen binding (green dashes) or hydrophobic (red semi-circle radial spikes) interaction with SIM binding mode in the crystal structure of SIM-HMGCR complex (bright blue). The peptide chains of HMGCR in 3D views are shown in green. The NADP is shown in pink and cis-loop cavity is shown in yellow

Figures 4a, 4d and 4g demonstrate the results of docking analysis done by Pymol in the 3D view; Figures 4b, 4e and 4h show the results of Ligplot diagram (in a 2D view), prepared by Ligplot plus); and Figure 4c, 4f and 4i are in superposition views, and were prepared by Pymol. Figures 4a, 4b and 4c show the binding site of SIM in the HMGCR, obtained through the computational survey of “the complex of the catalytic portion of human HMGCR X-ray crystal structure with simvastatin” (PDB ID: 1HW9). Figure 4a shows that SIM (cyan color) occupied the HMG-COA binding pocket (yellow) in HMGCR structure (green). Figure 4b shows that SIM was stabilized via five hydrogen bonds with amino acid residues: Ser684, Lys 691, Arg590, Asn755, and with hydrophobic interactions with 10 amino acid residues in the enzyme active site. Figure 4c demonstrates SIM spatial location, proportion to NADPH (pink) and cis-loop. To optimize and confirm the computational analysis, we did the docking of SIM-HMGCR interaction. Figures 4d to 4f show the results of the docking. Figure 4e shows that the SIM conformer obtained from docking analysis was stabilized on HMGCR active site via hydrogen bonding and hydrophobic interactions with 6 and 10 amino acid residues, respectively. Table 1 presents the amino acids involved in hydrogen bonding and hydrophobic interaction with SIM in crystal structure (Istvan and Deisenhofer, 2001), or SIM in the conformer obtained from docking analysis. The table reveals that 4 out of 4 and 8 out of 10 amino acid residues are involved in the hydrogen bonding and hydrophobic interactions of SIM with HMGCR, respectively. Figure 4f shows the superposition of SIM conformer with SIM in the crystallography data. These figures and Table 1 indicate that the docking data is completely consistent with the crystal structure, and the accuracy of docking analysis. Furthermore, based on Table 1, the binding energy of SIM interaction with HMGCR was -6.0 kcal/mol.

**Table 1:** Binding energy and amino acid residues involved in the interaction between different ligands with human HMGCR

Ligand	Amino acids involved in van der Waals binding	Amino acids involved in hydrogen bonding	ΔG (kcal/mol)
SIM^1^	Asp690, Leu853, Lys692, Leu562, Glu559, Cys561, Ser565, Ala751, *Lys735, Leu857*	Ser684, Lys 691, Arg590, Asn755	-----
SIM^2^	Asp690, Leu853, Lys692, Leu562, Glu559, Cys561, Ser565, Ala751, *His752, Leu853*	Ser684, Lys 691, Arg590, Asn755, *Lys692, Lys735*	-6.0
Crt	Asp690, Leu853, Glu559, Cys561, Ser565 *Ala564, Arg568, Ala856, Asn755, His752 *	Ser684	-6.6

SIM^1^: The parameters of simvastatin (SIM) binding with human HMGCR as determined by X-ray crystallography.

SIM^2^: The parameters of SIM binding with the crystal structure of human HMGCR after docking.

The bold and italic styles are representative of the similar and dissimilar amino acids involved in the binding of SIM^1^/SIM^2^ and Crt with HMGCR.

Figures 4g to 4i show the results of Crt docking with HMGCR X-ray crystal structure. Figures 4g and 4h show that Crt was stabilized in the HMG-COA binding pocket via hydrogen bonding of carboxyl moiety with Ser684, and hydrophobic interactions with 10 other amino acid residues. Table 1 shows that the only amino acid involved in hydrogen bonding and 5 out of 10 residues involved in the hydrophobic interaction with Crt were similar to those involved in the SIM interaction with HMGCR crystal structure. Superposition of Crt and SIM in HMGCR structure in Figure 4i shows the overlapping of some portions of these molecules, including the carboxyl moiety of Crt and HMG-like moiety of SIM. Table 1 shows that the binding energy of Crt interaction with HMGCR was -6.6 kcal/mol.

The data of Cro docking with crystal structure of HMGCR indicated that Cro failed to bind the active site of the enzyme (data not shown). Its mechanism of actions is possibly different from that of Crt and should be studied in the future.

## Discussion

Here, we showed that saffron-derived isoprenoids, Crt and Cro significantly reduced cholesterol and triglyceride levels in the serum and tumor tissues of mice with 4T1-induced breast cancer. Crt and Cro showed the same effect on the cytosolic lipids in MDA-MB-231, as a TNBC, and MCF-7, as a non-TNBC, cell line.

The MTT assay was performed to obtain the IC50 of Crt and Cro in MDA-MB-231 and MCF7 breast cancer cell lines. Then, these concentrations were used to treat these cell lines for further studies. The assay indicated that the IC50 of Cro against MDA-MB-231 and MCF7 was 4 mg/ml (4.09 mM) and 3.5 mg/ml (3.58 mM), respectively; the IC50 of Crt against these cell lines was 0.7 mg/ml (2.13 mM) and 0.8 mg/ml (2.43 mM), respectively. We previously published a review and showed that the IC50 values of Crt and Cro were respectively reported to be 2 μM-0.6 mM and 2 μM-5.5 mM in various cancer cell lines (Bolhassani et al., 2014 ▶). These differences are possibly due to the nature and genetic pattern of the cancer cells, the source and purity of Crt and Cro, and the experimental conditions of different laboratories.

Afterwards, we investigated the effects of Crt/Cro on lipid profile in the cytosol of the mentioned cell lines, for up to 24 h. As the statistical analysis indicated, there were significant differences between all treatment groups and the control group at different times; but, there were no significant differences between the different times. It indicated that most of the changes in the lipid content in the cells occurred up to 6 h and after that, slight changes were observed in these parameters.

Our data showed that both Crt and Cro reduced Chl and TG levels in the serum and tumor tissue of breast cancer bearing mice. These lipid-lowering effects of Crt/Cro in breast cancer were similar to those reported by us in diabetic and diabetic-atherosclerotic rats (Mahdavifard et al., 2016 ▶; Shirali et al., 2013 ▶). Application of Crt in the animal model of atherosclerosis also produced similar effects (Gainer and Jones, 1975 ▶). It was reported that Crt reduced the levels of total Chl, low density lipoprotein (LDL)-Chl and triglyceride in the serum, and inhibited the aortic plaque formation at the 9^th^ week after treatment of the atherosclerotic quails (He et al., 2007 ▶). A significant decrease in the levels of TG and LDL-Chl was also reported in hyperlipidemic mice after Crt treatment. This effect was comparable with lovastatin (Lee et al., 2005 ▶). Various mechanisms for these effects were suggested; They include, the inhibition of pancreatic and gastric lipases by Crt/Cro (Lee et al., 2005 ▶), the antioxidant effect of Crt (He et al., 2007 ▶) and the induction of glyoxalase system activity by Crt (Mahdavifard et al., 2016 ▶). We recently showed changes in the catalase activity *in vitro* and *in vivo* due to Crt and Cro treatment (Hashemi et al., 2019 ▶). We suppose that the hypolipidemic effect of Crt/Cro in the serum of tumor bearing mice and in the breast cancer cells, as we reported in the present study, was mediated by similar mechanisms. However, the exact mechanism should be studied in future.

The importance of lipids in tumor progression, invasion and metastasis has been described by some research groups (Kimura and Sumiyoshi, 2007 ▶; Luo et al., 2017 ▶). It was also shown that inhibition of cholesterol synthesis by lovastatin reduced tumor metastasis and migration in a mice model of mammary tumor (Alonso et al., 1998 ▶). In addition, targeting the HMGCR by ectopic expression of microRNA-195 not only reduced cellular cholesterol and lipid synthesis, but also inhibited metastasis and invasion in both TNBC (MDA-MB-231) and non-TNBC (MCF-7) cell lines (Singh et al., 2015 ▶). Thus, the hypolipidemic effects of both Crt and Cro are very important in the treatment and prevention of cancer progression, metastasis and invasion.

A mechanistic study showed that Cro reduced the lipid content of HepG2 cells under oleate treatment, in a more effective manner than SIM. However, immunohistochemical study indicated no significant effect of Cro on the HMGCR protein content in these cells (Leng et al., 2018 ▶). Since, there is no data about the effect of Crt or Cro on the HMGCR activity, we tried to predict possible interactions between Crt/Cro and HMGCR. The results of the docking analysis showed that Crt binds the active site of HMGCR with a negative free energy of -6.6 kcal mol^-1^. Visualizing the interaction by Pymol and Ligplot plus software showed that Crt occupies the cis-loop in the HMGCR, which is considered the HMG-COA binding pocket (Istvan and Deisenhofer, 2001 ▶; Tabernero et al., 2003 ▶). Consistently, superposition of Crt binding conformer and SIM binding mode in the crystal structure of HMGCR indicated the spatial overlapping of Crt with SIM. Previous studies showed that the competitive binding of ligands to HMG-COA binding pocket is the molecular basis of HMGCR inhibition by these ligands (Endo et al., 2004 ▶; Endo et al., 1977 ▶; Istvan and Deisenhofer, 2001 ▶; Tabernero et al., 2003 ▶). Therefore, it can be suggested that the molecular mechanism of Crt hypolipidemic effect in breast tumors and both breast cancer cell lines studied here, is possibly mediated through its interaction with HMGCR active site and competitive inhibition of the enzyme. In contrast to Crt, our docking analysis indicated that Cro binds a site far from the active site of HMGCR. Therefore, it may inhibit this enzyme through a non-competitive or allosteric inhibition. Kinetic studies should be done in future about the inhibitory effect of both Crt and Cro on HMGCR activity.

In conclusion, the results of the present study indicated that both Crt and Cro reduced the lipid content of the serum and breast tumors of 4T1-induced breast cancer mice. In a similar manner, these carotenoids decreased the lipid contents of MDA-MB-231 (a TNBC) and MCF-7 (a non-TNBC) breast cancer cell lines. In addition, the docking data indicated that Crt binds the active site of HMGCR with a free energy higher than SIM.
